
JOURNAL INFORMATION
==============================
NLM Title Abbreviation: Gastroenterol Rep (Oxf)
Journal ID: Gastroenterol Rep (Oxf)
NLM Journal ID: 101620508
Journal ID: gastro

Gastroenterology Report

EISSN: 2052-0034
Publisher: Oxford University Press

ARTICLE INFORMATION
==============================
PMCID: PMC5193067
DOI: 10.1093/gastro/gow038
Article ID: gow038
Article version: 1
Subjects: Chinese Abstracts

Chinese Abstracts – Volume 4 Issue 4*

*This article abstract was originally published in English. This translation has not been verified and should not be relied upon—it is provided for reference purposes only. The Publishers have not checked this translation and accept no liability for completeness or accuracy of this translation or the use of this translation for whatever purpose. This translation may be incomplete and inaccurate in whole or in part. If you need to rely upon a translation of this abstract, a professional human translator should be engaged to supply an accurate translation of the original English. When referencing articles from this journal, please always refer to the original English version, rather than a translated equivalent.

Print publication date: 2016 Nov
Electronic publication date: 2016 Nov 23
Volume: 4
Issue: 4
First page: C1
Last page: C3
Copyright: © The Author(s) 2016. Published by Oxford University Press and the Digestive Science Publishing Co. Limited.
Copyright year: 2016
License: This is an Open Access article distributed under the terms of the Creative Commons Attribution License (http://creativecommons.org/licenses/by/4.0/), which permits unrestricted reuse, distribution, and reproduction in any medium, provided the original work is properly cited.
License URL: https://creativecommons.org/licenses/by/4.0/

Page count: 3

==============================
OXFORD

*Gastroenterology Report*, 2016, 1–3

doi: 10.1093/gastro/gow038

### Chinese Abstracts – Volume 4 Issue 4*

### Review （综述）

### Care of inflammatory bowel disease patients in remission
### 缓解期炎性肠病患者的治疗

[摘要] 炎性肠病（IBD）包括溃疡性结肠炎和克罗恩病, 两者都系慢性复发性疾病， 具有较高的并发症发生率、病死率和治疗费用。鉴于社会上对慢性系统性疾病患者协同医疗的关注度不断提高， 本文重点讨论缓解期炎性肠病患者的医护， 包括患者的关切、生活质量、随访、首诊医生的作用及长期治疗方面的问题。我们通过PubMed对缓解期IBD患者进行广泛的文献检索， 并结合我们的自身经验， 撰写了这篇全面深入的综述。阐述了缓解期IBD患者所面临的难题， 包括（但不局限于）学习就业、社会心理、医疗保险、营养、生育和感染等问题。还特别关注了治疗策略的更新、吸烟对IBD的影响以及疫苗重要性等问题。缓解期IBD由于其多样性和复杂性, 治疗上极具挑战, 但相信随着胃肠科医师与其他相关医务人员的协同应对, 上述一些问题是可以得到解决的。

关键词：炎性肠病；缓解期；长期治疗

### Malnutrition: laboratory markers vs nutritional assessment
### 营养不良：实验室指标*vs*营养评估

[摘要] 营养不良是患者并发症发生率和病死率的一项独立危险因素，并造成医疗费用的增高。然而, 由于对营养不良没有一个统一的定义, 也没有一个标准的营养不良筛查/诊断方法， 导致全世界各地的医生对营养不良在认识上十分混乱， 各地的临床实践也有着各不相同的标准。作为营养不良的危险因素, 炎症的作用近期已被证实。组织学上, 一些血清蛋白如白蛋白和前白蛋白被广泛用于监测患者的营养状态。然而， 目前的研究热点是采用恰当的营养状态体格检查（NFPE）来诊断营养不良。目前认为，实验室指标本身不太可靠，只能作为NFPE的一个补充。今后的研究中需要筛选出一些诊断营养不良的血清生物学标志物， 这些标志物必须是不受炎症状态影响且具有无创及价格相对低廉的优势。然而， 一个完全的NFPE在营养不良的诊断中具有无可替代的作用。

关键词：营养不良；体格检查；血清标志物

### Original article （论著）

### An open-access endoscopy screen correctly and safely identifies patients for conscious sedation
### 开放获取内镜筛查能准确安全地筛选出可清醒镇静的病例

[摘要] 背景与目的：开放获取内镜筛查能很好地应用于普通低风险的内镜操作。内镜操作前的筛查重点是镇静需求， 但筛查结果欠准确将有损内镜的安全性。本研究旨在论证我们的开放获取内镜筛查系统能否准确筛选出可清醒镇静的病例。

方法：前瞻性连续入组经开放获取筛查系统或在胃肠科办公室咨询后进行预约的门诊内镜操作病例。收集错误预约为清醒镇静的病例， 对其病例特征进行分析。

结果：共计有8063例门诊病例预约行清醒镇静状态下的内镜操作, 其中5959例通过开放获取系统进行预约。仅78例（0.97%， 78/8063）后续被认为需要麻醉医生支持下的镇静处理, 其中44例（56.4%, 44/78）是通过开放获取筛查系统预约的， 长期服用鸦片（47.7%， 21/44）和苯二氮（34.1%， 15/44）是需要麻醉医生支持镇静的主要原因。长期服用苯二氮者较未服用者在清醒镇静时需要更高剂量的咪达唑仑（P=0.03）， 长期服用鸦片者镇静时则需要更高剂量的芬太尼（P=0.04）。进展期肝病和长期饮酒是胃肠科办公室咨询后预约病例需要麻醉医生支持镇静的常见原因, 其所占比例明显高于开放获取筛查系统预约病例（均P<0.01）。

结论：我们观察到经过多层筛查， 绝大多数病例都适合于清醒镇静, 更重要的是， 只有很少一部分（<1.0%）病例清醒镇静预约不当。需要麻醉医生辅助镇静的主要原因包括鸦片和苯二氮的长期服用和/或长期饮酒和进展期肝病, 提示这些病例应纳入开放获取筛查系统中。

关键词：开放获取内镜；镇静；筛查

### Risk factors and clinical implication of superimposed Campylobacter jejuni infection in patients with underlying ulcerative colitis
### 溃疡性结肠炎患者空肠弯曲杆菌重复感染的危险因素及临床意义

[摘要] 背景与目的：空肠弯曲杆菌重复感染（CJI）在溃疡性结肠炎（UC）患者中已有描述, 但其危险因素及对UC病程的影响尚不清楚。本研究旨在评估UC患者CJI的危险因素以及CJI对UC临床结局的影响。

方法：918例UC患者接受了空肠弯曲杆菌的检测, 其中21例（2.3%）发现CJI阳性（研究组）。对照组由84例年龄匹配的CJI阴性UC患者组

*This article abstract was originally published in English. This translation has not been verified and should not be relied upon—it is provided for reference purposes only. The Publishers have not checked this translation and accept no liability for completeness or accuracy of this translation or the use of this translation for whatever purpose. This translation may be incomplete and inaccurate in whole or in part. If you need to rely upon a translation of this abstract, a professional human translator should be engaged to supply an accurate translation of the original English. When referencing articles from this journal, please always refer to the original English version, rather than a translated equivalent.
本文摘要最初是以英文发表。中文摘译未获认证，谨供参考。出版社没有对中文摘译进行核验，不负责译文的完整性和准确性，也不会将其用以其他目的。中文摘译可能会出现部分不完整或不准确的情况。如果你需要准确获悉摘要，最好能请一位专业译者针对英文原文进行翻译。阅读杂志文章，请参考英文原文，不推荐中文翻译。

成。分析CJI的危险因素, 并对两组患者CJI诊断后1年的UC相关临床结局进行比较。

结果：10例（47.6%）患者在CJI诊断时需要入院治疗， 其中8例治疗是针对UC发作。经抗生素治疗后，13例（61.9%）CJI患者症状得以改善。多因素分析显示， 细菌检测前1年内曾住院治疗是CJI的独立危险因素（OR=3.9, 95%CI：1.1－14.1），而合并慢性肝病也可能是CJI的另一危险因素（OR=5.0，95%CI：0.92－8.3）。随访1年，研究组UC相关的结肠切除的比例（28.8% *vs* 14.3; *P*=0.11）和病死率（9.5% *vs* 1.2%; *P*=0.096）均有增高的趋势。

结论：近1年内住院治疗可致UC患者CJI风险增高。合并CJI的UC患者临床结局似乎更差, 常因UC发作接受入院治疗。

关键词：弯曲杆菌感染；溃疡性结肠炎；危险因素；临床结局

### Outcomes in children with Clostridium difficile infection: results from a nationwide survey

### 难辨梭菌感染患儿的结局：一项全国性调查结果

[摘要] 目的：基于医院和人群的研究都发现, 难辨梭菌感染（CDI）的发生率在成人与儿童中均不断上升, 但儿童CDI的结局仍不甚明确。我们分析了美国国家出院调查（NHDS）数据, 以了解住院患儿CDI情况。

方法：提取2005－2009年间的NHDS数据（包括人口统计资料、诊断情况和出院状态），应用ICD-9代码的诊断标准来筛选病例。采用加权单因素和多因素分析来明确CDI发生率及CDI与临床结局[包括住院时间、结肠切除术、各种原因导致的住院病死率及出院转至康复机构（DTCF）]的关系。

结果：在13.8百万住院患儿中有46 176例出现CDI，中位年龄为3岁，总发生率为33.5/万。CDI的实际发生率在2005－2009年间并没有明显变化（0.24%－0.43%, *P*=0.64）。单因素分析显示, CDI患儿中位住院时间延长（6 d *vs* 2 d），肠切除率（OR=2.0, 95%CI：1.7－2.4）、病死率（OR=2.5， 95%CI：2.3－2.7）和DTCF率（OR=1.6， 95%CI：1.6－1.7）增加（均*P*<0.0001）。按年龄、性别和术前合并症校正后，CDI是长住院时间（校正平均差6.4 d, 95%CI：5.4－7.4 d）、高肠切除率（OR=2.1，95%CI：1.8－2.5）、高病死率（OR=2.3，95%CI：2.2－2.5）和高DTCF率（OR=1.7，95%CI：1.6－1.8）的独立且最强的影响因素。排除婴儿后进行亚组分析, 结果显示, 与未出现CDI的儿童相比, CDI儿童的病死率和DTCF率显著增高, 住院时间亦显著延长。

结论：尽管对CDI认识不断提高, 治疗措施也在不断进步, CDI仍然是一个重要的临床问题, 会导致住院患儿住院时间延长, 肠切除率、住院病死率和DTCF率增高。

关键词：难辨梭菌感染；儿童；儿科患儿；临床结局；流行病学

### Association between small intestinal bacterial overgrowth and deep vein thrombosis

### 小肠细菌过度生长与深静脉血栓形成的关系

[摘要] 目的：小肠细菌过度生长（SIBO）会导致一些疾病。SIBO与深静脉血栓形成（DVT）的关系尚无报道。本研究旨在调查SIBO患者DVT的发生率及危险因素。

方法：收集Cleveland Clinic胃肠动力实验室数据库2008－2014年间接受葡萄糖氢/甲烷呼吸试验（诊断SIBO）和多普勒超声检查（诊断可疑DVT）的321例患者纳入研究，排除插管引起的DVT病例。主要研究指标是本组患者DVT的发生率和危险因素（包括SIBO）。

结果：本组321例入组患者中144例（44.9%）检测出SIBO, 53例（16.5%）经超声检查发现DVT。201例患者在DVT评估之前进行SIBO评估，呼吸试验至超声检查的中位时间为27月（四分位间距：11.0－45.0月）；120例在DVT评估之后进行SIBO评估， 超声检查至呼吸试验的中位时间为30月（四分位间距：11.8－54.3月）。单因素分析显示， DVT的发生与血栓栓塞事件家族史（35.8% *vs* 16.0%，*P*=0.001）、慢性肾病（26.4% *vs* 16.013.4%, *P*=0.019）和SIBO（69.8% *vs* 39.9%，*P*<0.001）显著相关。多因素分析显示, 血栓栓塞事件家族史（OR=3.39, 95%CI：1.67－6.8, *P*<0.001）、慢性肾病（OR=2.23，95%CI：1. 04－4.74，*P*=0.037）和SIBO（OR=3.27, 95%CI：1.70－6.32, *P*<0.001）可独立预测DVT的发生。

结论：SIBO与DVT的发生密切相关, 这种关系值得进一步研究。

关键词：小肠细胞过度生长；深静脉血栓形成；危险因素

### Expression of p53 predicts risk of prevalent and incident advanced neoplasia in patients with Barrett's esophagus and epithelial changes indefinite for dysplasia

### P53表达可预测上皮改变类型不确定的Barrett食管患者进展型腺瘤的罹患风险和进展风险

[摘要] 背景与目的：Barrett食管（BE）患者发生食管腺癌的风险增高, 因此, 需要常规内镜监测。如果上皮改变无法明确界定为阴性或阳性, 建议诊断为不确定型上皮改变（IND）。一些生物学标志物被认为可用以预测BE患者的瘤变, 但其在BE-IND患者是否具有同样的意义尚不清楚。因此， 我们通过回顾性研究来探讨这些生物学标志物是否可以预测BE-IND患者的瘤变。

方法：收集1992年1月至2007年12月间我中心所有BE-IND患者的临床资料。采用免疫组化分析来对P53、AMACR和cyclin D1的表达进行半定量。对罹患腺瘤/进展型腺瘤（首次随访中发现的病变）和新发腺瘤/进展型腺瘤（首次随访未发现、但在后续随访中发现的病变）的预测因素进行单因素分析。

结果：在纳入研究的103例BE-IND患者中，81例（78.6%）在诊断后12月内接受了1次内镜活检， 其中10例（12.3%）罹患腺瘤， 包括4例（4.9%）进展型腺瘤。在首次随访未发现腺瘤且后续随访超过1年的79例患者中，发现新发腺瘤18例（22.8%），其中4例（5.1%）为进展型腺瘤。AMACR和cyclin D1表达水平与罹患腺瘤或新发腺瘤均无关，但P53高表达（>5%）与罹患进展型腺瘤相关（P=0.04），且可增加后续进展型腺瘤的发生风险（HR=12, P=0.03）。

结论：本研究发现P53表达是BE-IND患者罹患及新发进展型腺瘤的预测标志物。

关键词：Barrett食管；瘤变；食管腺癌

### Long-term esophageal and respiratory outcomes in children with esophageal atresia and tracheoesophageal fistula

### 食管闭锁和气管食管瘘患儿的长期结局

[摘要] 目的：评估食管闭锁合并（或不合并）气管食管瘘（EA/TEF）患儿的长期（不局限于儿童时期）并发症和临床结局。本研究旨在通过临床症状、诊断试验和治疗干预评估一家三甲医院EA/TEF患儿吞咽和呼吸系统并发症。

方法：纳入2011－2014年间先天性EA/TEF患儿， 收集这些患儿的人口统计特征、疾病分类和分型、临床症状、影像学、内镜下及支气管镜下发现及其治疗情况。

结果：共计43例患儿纳入研究, 其中以白人居多, 中位年龄8岁（四分位间距320岁）。20例（62.5%, 20/32）为C型异常（EA合并远端TEF）。21例（48.8%）有烧心症状，19例（44.1%）有反酸症状，31例（72.1%）吞咽固体食物困难。26例患儿接受钡餐检查， 其中17例（65.4%）狭窄，20例（76.9%）动力不足，4例（15.4%）出现复发瘘。30例患儿接受上消化道内镜检查，其中21例（70.0%）发现狭窄，6例（20.0%）出现需外科干预的复发瘘。8例患儿行胃底折叠术。呼吸功能评估显示31例（72.1%）患儿有咳嗽和气梗，32例有呼吸困难和哮喘，19例（44.2%）患儿有反复的呼吸系统感染。其他发现包括气管软化（86.7%）和限制性肺病（54.5%）。

结论：EA/TEF患儿残留的食管和气管具有较高的病理风险， 需要长期随访来监测临床症状和治疗反应。

关键词：食管闭锁；气管食管瘘；胃食管反流病；食管动力不足；吸入性肺炎

### Association of conventional haemostasis and coagulation tests with the risk of acute upper gastrointestinal bleeding in liver cirrhosis: a retrospective study
传统止血和凝血试验与肝硬化急性上消化道出血的关系：一项回顾性研究

[摘要] 目的：回顾性比较伴与不伴急性上消化道出血（AUGIB）或急性食管静脉曲张出血（AEVB）的肝硬化患者止血/凝血指标的差异，包括血小板计数（PLT）、凝血酶原时间（PT）、国际标准化比率（INR）和活化部分凝血活酶时间（APTT）。

方法：2012年1月至2014年6月间，共计1734例肝硬化患者纳入研究。根据其病史分为AUGIB组（497例）和无AUGIB组（1237例）；同时根据内镜检查结果分为AEVB组（297例）和无AEVB组（1259例），另178例AUGIB患者由于缺乏内镜资料，既未入AEVB组，也未归入无AEVB组。

结果：AUGIB组与无AUGIB组患者相比，PLT（99.99±89.90 *vs* 101.47±83.03，*P*=0.734）和APTT（42.96±15.20 *vs* 43.77±11.01，*P*=0.219）的差异无统计学意义，但PT显著延长（17.30±5.62 *vs* 16.03±4.68，*P*<0.001），INR显著增高（1.45±0.69 *vs* 1.31±0.59，*P*<0.001）。PT可以独立预测AUGIB的发生，PT越低，AUGIB出现的可能性越小（OR＝0.968，95%CI：0.942－0.994）。AEVB组与无AEVB组患者相比，PLT显著减少（86.87±62.14 *vs* 101.74±83.62，*P*=0.004），APTT显著缩短（40.98±7.98 *vs* 43.72±10.97，*P*<0.001），但PT（16.53±3.71 *vs* 16.04±4.68，*P*=0.088）和INR（1.35±0.41 *vs* 1.31±0.59，*P*=0.225）的差异则无统计学意义。PLT可独立预测AEVB的发生，PLT越高，AEVB出现的可能性越大（OR＝1.004，95%CI：1.002－1.006）。

结论：PLT与肝硬化患者门脉高压出血密切相关。

关键词：凝血；出血；肝硬化；血小板；凝血酶原

### The presence of primary sclerosing cholangitis in patients with ileal pouch anal- anastomosis is associated with an additional risk for vitamin D deficiency
原发性硬化性胆管炎会进一步导致回肠储袋肛管吻合患者维生素D缺乏

[摘要] 目的：溃疡性结肠炎（UC）患者回肠储袋肛管吻合（IPAA）术后常出现维生素D缺乏。IPAA患者出现原发性硬化性胆管炎（PSC）后维生素D水平是否会进一步降低，目前仍不明了。本研究的目的是评估PSC是否是UC患者IPAA术后维生素D缺管的一个危险因素。

方法：在该病例对照研究中，74例出现PSC的IPAA患者纳入研究组，79例未出现PSC的IPAA患者作为对照组。44项指标被纳入多因素逻辑回归模型中进行分析。

结果：153例患者入组研究，其中研究组74例（48.4%），对照组79例（51.6%）。研究组维生素D水平是（18.9±1.4）ng/dL，明显低于对照组的（30.3±1.7）ng/dL（P＝0.011）。共有65例（42.5%）患者出现维生素D缺乏，其中49例（75.4%）合并PSC。多因素分析显示，PSC的出现（OR＝7.56，95%CI：2.29－24.08；P＝0.001）和维生素D的补充（OR＝2.58，95%CI：1.57－9.19；P＝0.018）与维生素D缺乏密切相关。

结论：PSC的出现是UC患者IPAA术后维生素D缺乏的独立危险因素。这些患者应该被筛查出来，以对其维生素D水平进行严密监测。

关键词：炎性肠病；原发性硬化性胆管炎；回肠储袋肛管吻合术；维生素D缺乏

### Case Report（病例报告）

### Spontaneous closure of stoma
肠造口的自行闭合

### A rare case of infectious colitis
一例罕见的感染性结肠炎

### Case report of a traumatic rectal neuroma
外伤性直肠神经瘤一例报道

### Computed tomography-guided endoscopic needle knife therapy for ileal pouch sinus
CT导向的内镜下针刀治疗回肠储袋窦道

### Jejunal diverticulosis found in a patient with long-standing pneumoperitoneum and pseudo-obstruction on imaging: a case report
表现为长期影像学气腹和假性梗阻的空肠憩室一例报道
